# Overexpression of CDC7 in malignant salivary gland tumors correlates with tumor differentiation^☆^

**DOI:** 10.1016/j.bjorl.2017.11.004

**Published:** 2017-12-26

**Authors:** Zohreh Jaafari-Ashkavandi, Mohammad Javad Ashraf, Ali Asghar Abbaspoorfard

**Affiliations:** a Shiraz University of Medical Sciences, School of Dentistry, Department of Oral and Maxillofacial Pathology, Shiraz, Iran; b Shiraz University of Medical Sciences, School of Medicine, Department of Pathology, Shiraz, Iran

**Keywords:** Salivary gland, CDC7, Adenoid cystic carcinoma, Mucoepidermoid carcinoma, Pleomorphic adenoma, Glândula salivar, CDC7, Carcinoma adenoide cístico, Carcinoma mucoepidermoide, Adenoma pleomórfico

## Abstract

**Introduction:**

Cell division cycle-7 protein is a serine/threonine kinase that has a basic role in cell cycle regulation and is a potential prognostic or therapeutic target in some human cancers.

**Objectives:**

This study investigated the expression of cell division cycle-7 protein in benign and malignant salivary gland tumors and also its correlation with clinicopathologic factors.

**Methods:**

Immunohistochemical expression of cell division cycle-7 was evaluated in 46 cases, including 15 adenoid cystic carcinoma, 12 mucoepidermoid carcinoma, 14 pleomorphic adenoma, and 5 normal salivary glands. Cell division cycle-7 expression rate and intensity were compared statistically.

**Results:**

The protein was expressed in almost all tumors. The intensity and mean of cell division cycle-7 expression were higher in malignant tumors in comparison with pleomorphic adenomas (*p* = 0.000). The protein expression was correlated with tumor grades (*p* = 0.000).

**Conclusions:**

The present study demonstrated cell division cycle-7 overexpression in malignant salivary gland tumors in comparison with pleomorphic adenomas, and also a correlation with tumor differentiation. Therefore, this protein might be a potential prognostic and therapeutic target for salivary gland tumors.

## Resumo

**Introdução:**

A *cell division cycle-7* é uma serina/treonina quinase que tem um papel básico na regulação do ciclo celular e é um potencial marcador prognóstico ou terapêutico em alguns tipos de câncer humano.

**Objetivos:**

Este estudo investigou a expressão de *cell division cycle-7* em tumores de glândulas salivares benignos e malignos e também sua correlação com fatores clínico-patológicos.

**Método:**

A expressão imuno-histoquímica de *cell division cycle-7* foi avaliada em 46 casos, incluindo 15 carcinomas adenoide císticos, 12 carcinomas mucoepidermoides, 14 adenomas pleomórficos e 5 glândulas salivares normais. A taxa de expressão e a intensidade da proteína *cell division cycle-7* foram comparadas estatisticamente.

**Resultados:**

A proteína foi expressa em quase todos os tumores. A intensidade e a média da expressão de *cell division cycle-7* foram maiores em tumores malignos em comparação com adenoma pleomórfico (*p* = 0,000). A expressão da proteína foi correlacionada com os graus do tumor (*p* = 0,000).

**Conclusões:**

O presente estudo demonstrou a superexpressão de *cell division cycle-7* em tumores malignos de glândulas salivares quando comparada com o adenoma pleomórfico, além de uma correlação com a diferenciação de tumores. Portanto, essa proteína pode ser um potencial marcador prognóstico e terapêutico para tumores de glândulas salivares.

## Introduction

Salivary gland tumors (SGT) are relatively rare and diverse tumors which account for 3–6% of all head and neck neoplasms.^1^ These tumors consist of different benign and malignant subtypes with a wide histopathologic spectrum which may overlap with each other; however, with a different clinical behavior and management, pleomorphic adenoma (PA), mucoepidermoid carcinoma (MEC) and adenoid cystic carcinoma (AdCC) are the most common benign and malignant SGTs. Surgery is the main treatment and in malignant tumors adjuvant chemo-radiotherapy may be required. Today, there is much promise in finding novel anti-cancer treatments to the basis of molecular target-therapy. The key molecules that participate in the cell growth and division are promising candidates for this goal. They may affect a broad range of various tumor types with a high proliferation rate.^2^

Cell division cycle-7 protein (CDC7) is a serine-threonine kinase, originally introduced in budding yeast, and plays a key role in DNA replication, principally by activating MCM complex, and regulation of S-phase check point in the cell cycle.^3^, ^4^ The regulator subunit of CDC7 is Dbf4/activator of S-phases.^5^ CDC7 overexpression was also correlated with P53 inactivation^5^ and has been found in many human tumor cell lines and tissues, including breast, colon and lung cancers, melanoma and oral squamous cell carcinoma (OSCC), but this protein has very low or undetectable expression in normal tissues.^2^, ^5^, ^6^, ^7^, ^8^, ^9^, ^10^

It has been shown that CDC7 overexpression was correlated with poor prognosis in patients with B-cell lymphoma.^11^ Also, it contributed to the resistance to DNA damaging agents and increasing survival in OSCC cancer cell line.^9^ CDC7 was a therapeutic target in ovarian carcinoma.^12^ Therefore, CDC7 is a promising and potent candidate target for cell-growth inhibition because it points DNA replication before it starts.^4^, ^6^, ^13^ Therefore, evaluation of CDC7 function in any specific tumor is suggested. To the best of our knowledge, there is no research focused on CDC7 expression and its significance in SGTs. This study aimed to evaluate CDC7 expression rate and its correlation with clinicopathologic parameters of the most common benign and malignant SGTs.

## Methods

### Tissue samples

In this cross-sectional retrospective study, 46 cases consisting of 14 PA, 15 AdCC, 12 MEC and 5 normal salivary glands (NSG) were included. The cases obtained from archive of Pathology Department from 2009 to 2014. All cases had definitive diagnosis and adequate epithelial tissue. Severe inflamed cases were excluded. The baseline data including patient's age and gender, as well as tumor site, size, grade and stage were recorded, using the patient's medical files.

### Immunohistochemistry

4 μm tissue sections were provided from formalin-fixed and paraffin-embedded blocks. After deparaffinization and rehydration, antigen retrieval was performed by Tris-buffer in PH = 8 at 121 °C for 20 min. Endogenous peroxidase activity was blocked using 3% hydrogen peroxide for 30 min. Then, the sections were incubated by primary antibody (anti-CDC7 polyclonal antibody, 1:50, Genetex Company, USA) for 60 min. Envision system was applied as secondary antibody and the section were washed in PBS. The chromogen solution was 3,3′-diaminobenzidine tetrahydrochloride (DAB). Finally, the slides were counterstained with Mayer's hematoxylin. A section of normal lymph node was used as positive control and the same section by omitting primary antibody as the negative control.

The stained tissues evaluated by light microscopy and the cells with brown nuclei were considered as positive. In each case, at least 1000 cells were counted in 3 microscopic fields and the percentage of positive cells was noted. The intensity of staining was assessed and scored as 1 – mild or 2 – moderate/severe, in comparison with the positive control. The mean of CDC7 expression was scored as: (1) positive cells < 5%, (2) 5–10% and (3) >10%. The final score was obtained by multiplying the intensity and percentage scores. Data were analyzed by Kruskal–Wallis, Tukey and Dunn tests and Spearman's correlation. *p* < 0.05 was considered as significant.

## Results

The patients were 18 males and 28 females with a mean age of 49.4 ± 15. The baseline data related to all study groups are illustrates in Table 1.

**Table 1:** Basic information of all study groups.

Group (*n*)	PA (14)	MEC (12)	AdCC (15)	NSG (5)	Total (46)
Age	39.4 ± 14	60.6 ± 12	49.7 ± 12	49 ± 14	49.4 ± 15.1
(M/F)	5/9	7/5	4/11	2/3	18/28
Site (Major/Minor)	11/2	10/2	6/8	1/4	28/16
Grade (1, 2, 3)	–	3, 0, 8	5, 10, 0	–	8, 10, 8
Stage (1, 2, 3, 4)	–	1, 3, 2, 5	1, 3, 4, 5	–	2, 6, 6, 10
Size (1, 2, 3, 4)	–	1, 5, 2, 3	1, 4, 3, 5	–	2, 9, 5, 8

PA, pleomorphic adenoma; MEC, mucoepidermoid carcinoma; AdCC, adenoid cystic carcinoma; NSG, normal salivary glands.

Clinic data of some cases were not available.

In NSGs, two specimens exhibited a limited nuclear staining in acinar and ductal cells, with weak to moderate intensity. Almost all tumors, except one MEC, showed positive nuclear CDC7 expression.

**Figure 1:**
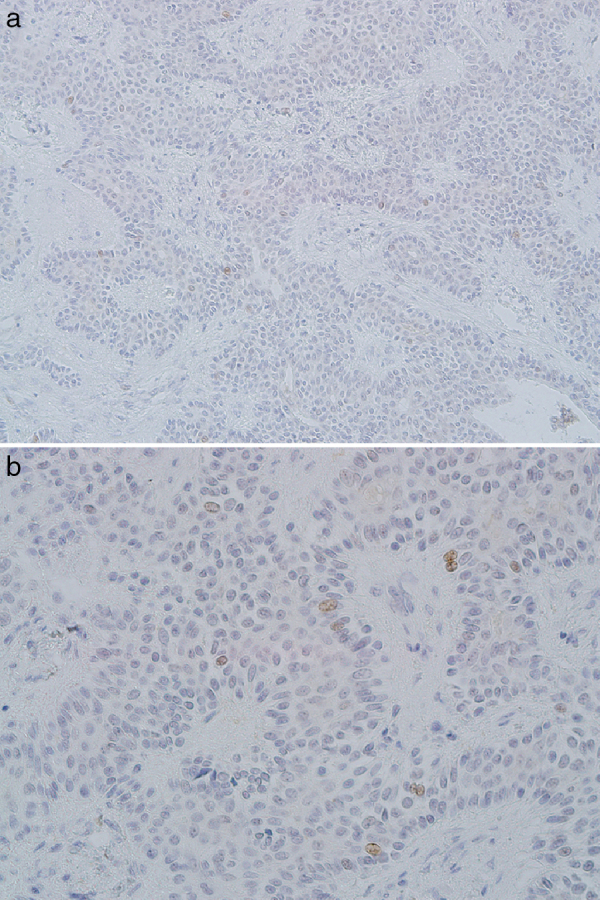
Nuclear, weak CDC7 expression in pleomorphic adenoma (A, ×200 and B, ×400).

PAs showed CDC7 expression in the epithelial and ductal cells (Fig. 1A and B) with a mean of 2.3 ± 1.2. 71% of the cases revealed weak staining.

**Figure 2:**
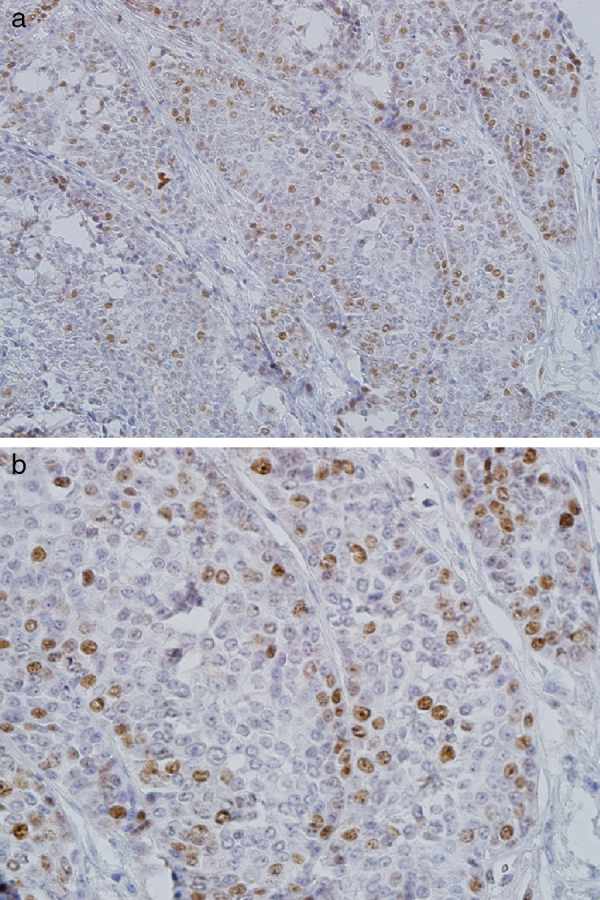
Severe nuclear CDC7 expression in epidermoid cells of high-grade mucoepidermoid carcinoma (A, ×200; B, ×400).

**Figure 3:**
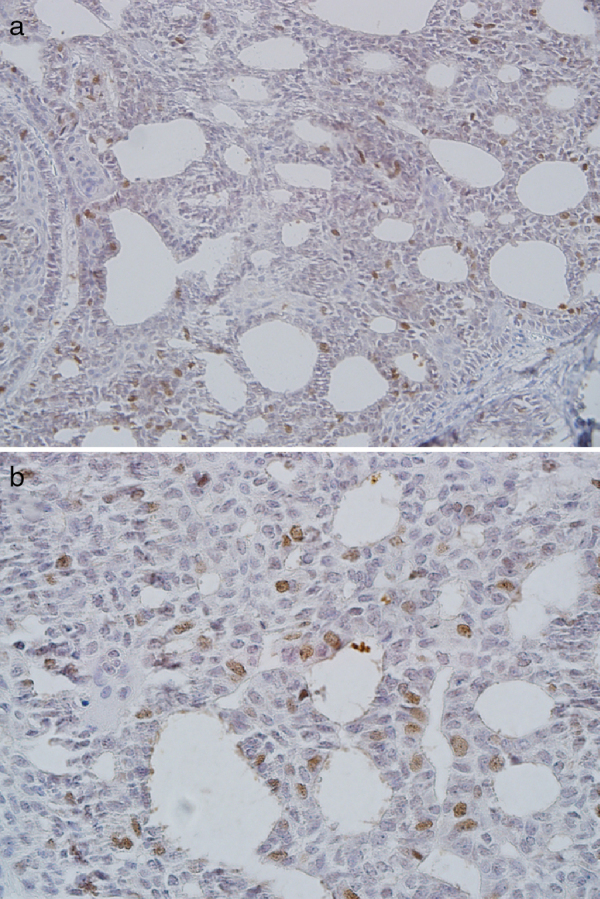
Severe nuclear CDC7 expression in adenoid cystic carcinoma (A, ×200; B, ×400).

The epidermoid cells of MEC showed CDC7 staining (Fig. 2A and B) and most of the cases (74%) had moderate/severe expression with a mean of 32.1 ± 14.3. Mucous and clear cells were not stained. All cases of AdCC exhibited moderate/severe CDC7 expression with a mean of 9.7 ± 3 (Fig. 3A and B).

Details about mean expression of CDC7, intensity of staining and final scores are shown in Table 2. Kruskal–Wallis test showed a significant difference among groups in CDC7 expression (*p* = 0.000). Dunn test showed this difference between PA and MEC, as well as PA and AdCC (*p* = 0.000 and *p* = 0.004). However, there was not any significant difference between MEC and AdCC groups. Also, final scores of the groups were significantly different, according to Kruskal–Wallis test (*p* = 0.000).

**Table 2:** Scores of intensity and CDC7 mean expression in all study groups.

	PA	MEC	AdCC	NSG
*CDC7 mean score*				
1	5	0	0	2
2	9	1	7	0
3	0	10	7	0

*Intensity score*				
1	10 (71.4)	2 (18.2)	0	0
2	4 (28.6)	9 (81.8)	15 (100)	2 (100)

PA, pleomorphic adenoma; MEC, mucoepidermoid carcinoma; AdCC, adenoid cystic carcinoma; NSG, normal salivary glands.

PA showed significant lower scores of CDC7 mean, in comparison with MEC and also with AdCC groups, using Dunn test (*p* = 0.000 and *p* = 0.02, respectively). However, MEC and AdCC groups were statistically similar (Dunn test, *p* = 0.26).

Final scores were also different among the groups according to the results of Kruskal– Wallis test (*p* = 0.000) (Table 3). Dunn test showed that finals scores of the PA group was significantly lower than both malignant tumors (*p* = 0.000); however, MEC and AdCC were not different (*p* = 1).

**Table 3:** Final scores of all study groups (Intensity × Mean scores).

Scores	1	2	3	4	6
MEC	1	0	2	0	9
AdCC	0	0	1	5	7
PA	0	7	0	2	0
NSG	0	2	0	0	0

Total	1	9	3	7	16

PA, pleomorphic adenoma; MEC, mucoepidermoid carcinoma; AdCC, adenoid cystic carcinoma; NSG, normal salivary glands.

High-grade tumors showed a significant increased CDC7 expression in comparison with low and intermediate grades. Spearman's correlation test showed that CDC7 expression was correlated with tumor grades (*p* = 0.000), but not with tumor stage or patient's age and gender (*p* > 0.05).

## Discussion

In the present study, we described CDC7 expression in PA, MEC and AdCC, and also its overexpression in malignant SGTs in comparison with benign ones and normal glands. Various studies have previously revealed that CDC7 has a basic role in cell proliferation, tumorogenesis and malignant progression^6^ by activating DNA replication.^5^, ^8^ Our findings support previous studies in various human cancer cell lines and tissues. Melling et al. demonstrated overexpression of CDC7 protein in colorectal cancer in association with P53 overexpression and as a favorable prognostic marker.^2^ Bonte et al. also showed that CDC7 is very low or undetectable in normal tissue, but it was over-expressed in the human breast, colon and lung cancer cell lines.^5^ One study showed increased CDC7 mRNA level in malignant transformed breast cancer cell line and also in hyper-proliferating cells in a lesser degree in comparison with primary normal cells.^6^ Increased CDC7 expression has been a marker of OSCC and has contributed to the resistance to DNA-damaging material.^9^ CDC7 and bf4 subunit form a complex that acts as an active protein kinase in all organisms.^14^ The main target of that complex activity is the MCM complex. The strong MCM positive cells indicate a high CDC7 activity.^11^ MCM2-7 have considered as replication initiation factors and, as a novel diagnostic and prognostic biomarker for several human cancers. MCM expression has been reported in the tumors that showed CDC7 overexpression such as OSCC, breast cancer cell line and tissue and also SGTs.^15^, ^16^, ^17^, ^18^ MCM2 and MCM3 represented an overexpression in malignant SGTs in comparison with benign ones^17^, ^18^ which indirectly support the overexpression of CDC7 in our samples.

It has also been demonstrated that CDC7 overexpression was correlated with TP53 gene mutation. CDC7 inhibition can induce cell death via a P53-dependent pathway.^19^ Bonte et al. found a correlation between P53 loss, and CDC7 overexpression in some cancer cell line.^5^ Also, in another study CDC7 expression was linked to P53 positivity in colorectal cancer.^2^ CDC7 was a therapeutic target for P53 mutant breast cancer.^6^ Previous studies on SGTs showed a higher expression of P53 in malignant SGTs in comparison with PA, which indirectly support our findings.^20^

The present study showed a positive correlation between CDC7 expression and tumor grades. However, our data did not show a significant difference between AdCC and MEC in CDC7 expression. Although AdCC is a high-grade tumor with more aggressive behavior in comparison with MEC, in our samples most of the MEC specimens were high-grade tumors. In agreement with our results, increased CDC7 expression was linked to loss of tumor differentiation, genomic instability and development of aggressive phenotype in breast cancer.^6^ Also, high grade colorectal and ovarian cancers showed higher CDC7 expression.^2^, ^12^ This association of CDC7 expression with differentiation makes this protein a potential suitable target for therapeutic and prognostic approaches. However, in contrast to these researches, our limited cases with complete data about clinical stage did not show any significant correlation with protein expression.

The present study revealed nuclear staining of CDC7 in all positive specimens which reinforced CDC7 function in DNA replication. Previous studies have demonstrated protein accumulation in the cell nuclei during G1 phase.^21^

## Conclusion

The present findings showed CDC7 expression in the most common benign and malignant SGTs and its overexpression in malignant ones. Positive correlation of this protein with tumoral differentiation may represent it as a potential prognostic and therapeutic target.

## Conflicts of interest

The authors declare no conflicts of interest.

## References

[1] Jaafari-Ashkavandi Z., Ashraf M., Afandak N. (2011). A clinico-pathologic study of 82 intraoral minor salivary gland tumors. I Red Crescent Med J.

[2] Melling N., Muth J., Simon R., Bokemeyer C., Terracciano L., Sauter G. (2015). Cdc7 overexpression is an independent prognostic marker and a potential therapeutic target in colorectal cancer. Diagn Pathol.

[3] Dally R.D., Woods T.A. (2014).

[4] Montagnoli A., Moll J., Colotta F. (2010). Targeting cell division cycle 7 kinase: a new approach for cancer therapy. Clin Cancer Res.

[5] Bonte D., Lindvall C., Liu H., Dykema K., Furge K., Weinreich M. (2008). Cdc7-Dbf4 kinase overexpression in multiple cancers and tumor cell lines is correlated with p53 inactivation. Neoplasia.

[6] Choschzick M., Lebeau A., Marx A.H., Tharun L., Terracciano L., Heilenkötter U. (2010). Overexpression of cell division cycle 7 homolog is associated with gene amplification frequency in breast cancer. Hum Pathol.

[7] Barkley L.R., Santocanale C. (2013). MicroRNA-29a regulates the benzo[a]pyrene dihydrodiol epoxide-induced DNA damage response through Cdc7 kinase in lung cancer cells. Oncogenesis.

[8] Clarke L.E., Fountaine T.J., Hennessy J., Bruggeman R.D., Clarke J.T., Mauger D.T. (2009). Cdc7 expression in melanomas, Spitz tumors and melanocytic nevi. J Cutan Pathol.

[9] Cheng A.N., Jiang S.S., Fan C.C., Lo Y.K., Kuo C.Y., Chen C.H. (2013). Increased Cdc7 expression is a marker of oral squamous cell carcinoma and overexpression of Cdc7 contributes to the resistance to DNA-damaging agents. Cancer Lett.

[10] Chen H.-J., Zhu Z., Wang X.-L., Feng Q.-L., Wu Q., Xu Z.-P. (2013). Expression of huCdc7 in colorectal cancer. World J Gastroenterol.

[11] Hou Y., Wang H.Q., Ba Y. (2012). High expression of cell division cycle 7 protein correlates with poor prognosis in patients with diffuse large B-cell lymphoma. Med Oncol.

[12] Kulkarni A.A., Kingsbury S.R., Tudzarova S., Hong H.-K., Loddo M., Rashid M. (2009). Cdc7 kinase is a predictor of survival and a novel therapeutic target in epithelial ovarian carcinoma. Clin Cancer Res.

[13] Ito S., Taniyami C., Arai N., Masai H. (2008). Cdc7 as a potential new target for cancer therapy. Drug News Perspect.

[14] Miller C.T., Gabrielse C., Chen Y.C., Weinreich M. (2009). Cdc7p-Dbf4p regulates mitotic exit by inhibiting Polo kinase. PLoS Genet.

[15] Jurikova M., Danihel L., Polak S., Varga I. (2016). Ki67, PCNA, and MCM proteins: markers of proliferation in the diagnosis of breast cancer. Acta Histochem.

[16] Razavi S.M., Jafari M., Heidarpoor M., Khalesi S. (2015). Minichromosome maintenance-2 (MCM2) expression differentiates oral squamous cell carcinoma from pre-cancerous lesions. Malays J Pathol.

[17] Vargas P.A., Cheng Y., Barrett A.W., Craig G.T., Speight P.M. (2008). Expression of Mcm-2, Ki-67 and geminin in benign and malignant salivary gland tumours. J Oral Pathol Med.

[18] Jaafari-Ashkavandi Z., Najvani A.D., Tadbir A.A., Pardis S., Ranjbar M.A., Ashraf M.J. (2013). MCM3 as a novel diagnostic marker in benign and malignant salivary gland tumors. Asian Pac J Cancer Prev.

[19] Vanotti E., Amici R., Bargiotti A., Berthelsen J., Bosotti R., Ciavolella A. (2008). Cdc7 kinase inhibitors: pyrrolopyridinones as potential antitumor agents. 1. Synthesis and structure-activity relationships. J Med Chem.

[20] Al-Rawi N.H., Omer H., Al Kawas S. (2010). Immunohistochemical analysis of P(53) and bcl-2 in benign and malignant salivary glands tumors. J Oral Pathol Med.

[21] Sato N., Sato M., Nakayama M., Saitoh R., Arai K., Masai H. (2003). Cell cycle regulation of chromatin binding and nuclear localization of human Cdc7-ASK kinase complex. Genes Cells.

